# Oseltamivir (Tamiflu), a commonly prescribed antiviral drug, mitigates hearing loss in mice

**DOI:** 10.1002/ctm2.1803

**Published:** 2024-08-12

**Authors:** Emma J. Sailor‐Longsworth, Richard D. Lutze, Matthew A. Ingersoll, Regina G. Kelmann, Kristina Ly, Duane Currier, Taosheng Chen, Jian Zuo, Tal Teitz

**Affiliations:** ^1^ Department of Pharmacology and Neuroscience School of Medicine Creighton University Omaha Nebraska USA; ^2^ Department of Chemical Biology and Therapeutics St. Jude Children's Research Hospital Memphis Tennessee USA; ^3^ Department of Biomedical Sciences School of Medicine Creighton University Omaha Nebraska USA; ^4^ The Scintillon Research Institute San Diego California USA

Dear Editor,

Hearing loss affects up to 10% of people worldwide and therapeutic interventions are desperately needed. Noise exposure and chemotherapy treatments are leading causes of this impairment, but currently there is only one FDA‐approved drug for a subgroup of cisplatin‐treated cancer patients.^1^, ^2^ Hearing loss can arise from damage to many different inner ear cell types with outer hair cell (OHC) and synaptic dysfunction as two of the most common aetiologies of hearing loss.^2^, ^3^, ^4^ Drug repurposing is a strategy for addressing unmet medical needs that can be quicker and more cost‐effective than traditional drug development.^3^ Here, we performed unbiased cell‐based screens of 1300 FDA‐approved drugs and tested our top candidate oseltamivir phosphate (brand name Tamiflu), a common influenza antiviral drug, in established cisplatin‐ and noise‐induced hearing loss animal models. Our results support oseltamivir as a promising otoprotective therapeutic candidate for both cisplatin chemotherapy and traumatic noise exposure.

Oseltamivir phosphate and its active form, oseltamivir carboxylate, protect against cisplatin‐induced hair cell loss in mouse cochlear explants without interfering with cisplatin's tumour killing efficacy in tumour cell lines. The prodrug, oseltamivir phosphate, tested at a dose of 3 µM, was a top hit in high‐throughput screens reducing 95% of the caspase‐3/7 cell death activity of cisplatin‐treated cells.^3^, ^4^ In mouse P3 cochlear explants, the prodrug, oseltamivir phosphate, protected from cisplatin‐induced OHC death with an EC_50_ of 450 nM (Figure 1A,D), while the active antiviral drug, oseltamivir carboxylate (Figure 1B), had a similar EC_50_ of 505 nM (Figure 1C,E). Importantly, oseltamivir cotreatment in three small cell lung carcinoma and three neuroblastoma cell lines did not interfere with cisplatin's ability to kill tumour cells (Figure 1F–K).

**FIGURE 1:**
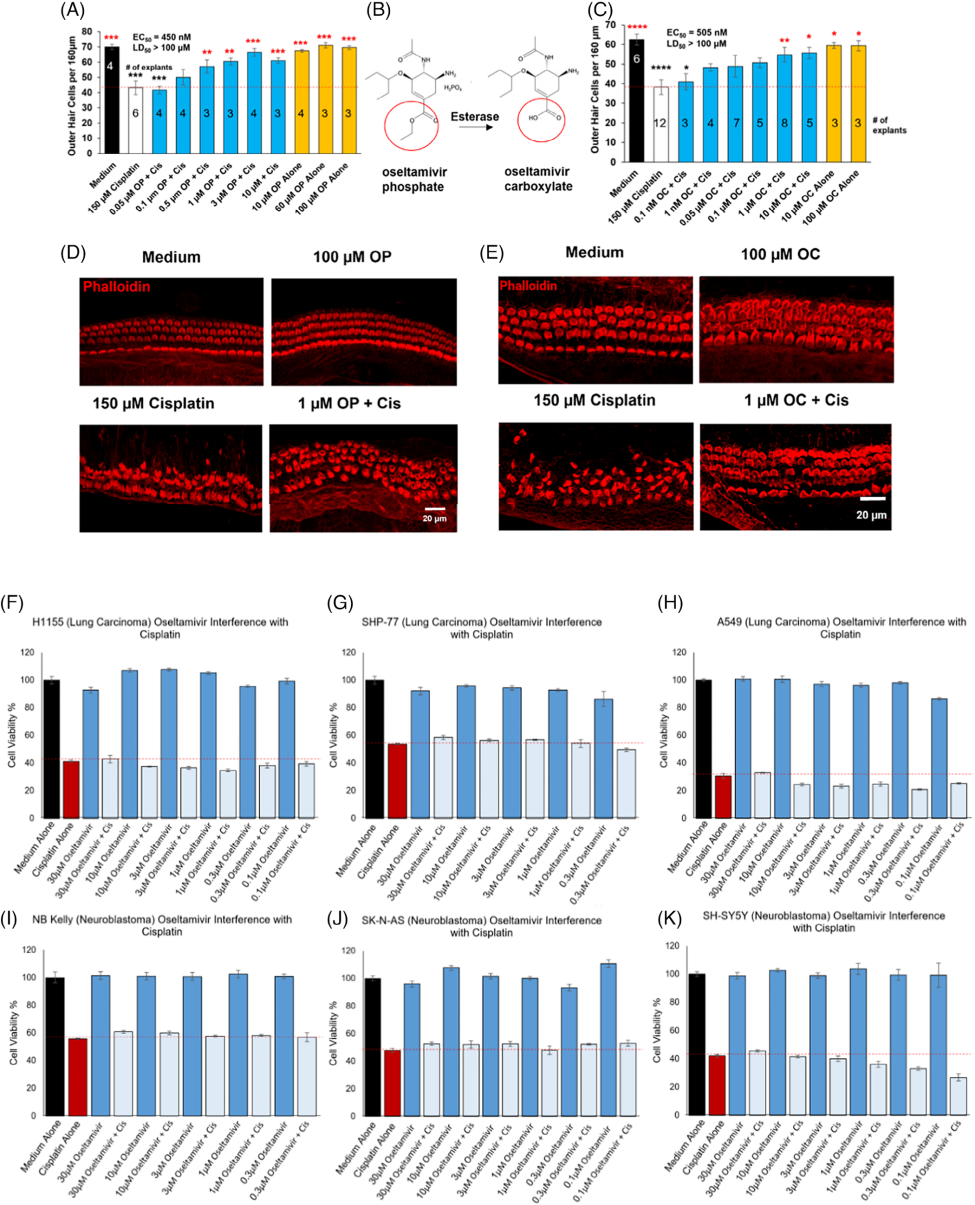
Oseltamivir, an antiviral neuraminidase inhibitor, reduces cisplatin‐induced cell death in murine cochlear explants and does not interfere with cisplatin's tumour killing ability in three lung carcinoma and three neuroblastoma cell lines. (A) Dose–response relationship of the pro‐drug, oseltamivir phosphate and outer hair cell (OHC) count in P3 FVB pup cochlear explants treated with cisplatin. Black: media alone; white: cisplatin alone; gold: oseltamivir phosphate alone; blue: cisplatin (150 µM) and oseltamivir phosphate (.05–10 µM) cotreatment. Cotreated samples were pretreated with oseltamivir phosphate for 1 h prior to cisplatin treatment, and then cultured in oseltamivir phosphate‐ and cisplatin‐treated media for 24 h. Numbers within bars denote number of explants per treatment. Samples were then stained with phalloidin with OHC counts per 160 µm taken from the cochlear middle turn. (B) Molecular structure of the prodrug, oseltamivir phosphate and its active antiviral metabolite, oseltamivir carboxylate. Hydrolysis of the circled ether group by hepatic esterase yields oseltamivir carboxylate, a sialic acid analogue and viral neuraminidase inhibitor. (C) Dose–response relationship of the metabolite oseltamivir carboxylate and OHC count in P3 FVB pup cochlear explants treated with cisplatin. Black: media alone; white: cisplatin alone; gold: oseltamivir carboxylate alone; blue: cisplatin (150 µM) and oseltamivir carboxylate (.001–10 µM) cotreatment. (D and E) Representative confocal images of explants treated with medium alone, 100 µM oseltamivir phosphate (D) or oseltamivir carboxylate (E), 150 µM cisplatin, and 3 µM oseltamivir phosphate (D) or 1 µM oseltamivir carboxylate (E) + 150 µM cisplatin. Data shown as means ± SEM, ^*^ *p* < .05, ^**^ *p* < .01, ^***^ *p* < .001 compared to cisplatin alone (red) and medium alone (black) by one‐way analysis of variance (ANOVA) with Bonferroni post hoc test. (F–K) Three neuroblastoma and three lung carcinoma cell lines were treated with cisplatin and six varying oseltamivir concentrations and then the Cell Titer‐Glo assay was performed to determine cell viability. Cell viability graphs after cisplatin and oseltamivir treatment for the (F) H1155, (G) SHP‐77, (H) A549, (I) Kelly, (J) SK‐N‐AS and (K) SH‐SY5Y cell lines are shown. Medium alone (black), cisplatin alone (red), oseltamivir alone (dark blue) and oseltamivir + cisplatin (light blue). Data shown as means ± SEM, compared to cisplatin alone by one‐way ANOVA with Bonferroni post hoc test. *n* = 6 wells.

Oseltamivir protects mice from cisplatin ototoxicity after a single, high dose of cisplatin and in a clinically relevant, multicycle cisplatin protocol. Adult FVB/NJ mice were treated orally with 50 mg/kg oseltamivir phosphate, 45 min before one dose of 30 mg/kg cisplatin (Figure 2A). We measured auditory brainstem response (ABR) as a test of hearing function. ABR measures nerve electrical activities from the cochleae to the brain. The ABR threshold is the lowest decibel sound pressure level (dB SPL) an animal can hear at. Mice cotreated with oseltamivir and cisplatin had 15 dB lower ABR threshold shifts at the 32 kHz region and displayed reduction in OHC death at the middle and basal cochlear regions compared to cisplatin‐treated mice (Figure 2B–E). Utilising a clinically relevant multicycle cisplatin mouse model that mimics cisplatin treatment in humans (Figure 2F),^4^, ^5^, ^6^ mice were treated orally with 50, 10 or 2 mg/kg oseltamivir, 45 min before the cisplatin treatment in the morning and 12 h later for three consecutive days. An amount of 3 mg/kg of cisplatin was administered via intraperitoneal injection. Mice cotreated with 50 and 10 mg/kg oseltamivir had significantly lower ABR threshold shifts at the 16 and 32 kHz regions, while 2 mg/kg oseltamivir treatment had no difference compared to cisplatin alone (Figure 2G,H). The 50 and 10 mg/kg oseltamivir cotreated mice with cisplatin had significantly higher ABR wave 1 amplitudes at 90 dB (Figure 2L), but no reduction in distortion product otoacoustic emission (DPOAE) threshold shifts (Figure 2I). The 50 and 10 mg/kg oseltamivir cotreatments conferred a significant reduction in OHCs loss (Figure 2J,K) and no significant difference in weight loss compared to cisplatin alone (Figure 2M).

**FIGURE 2:**
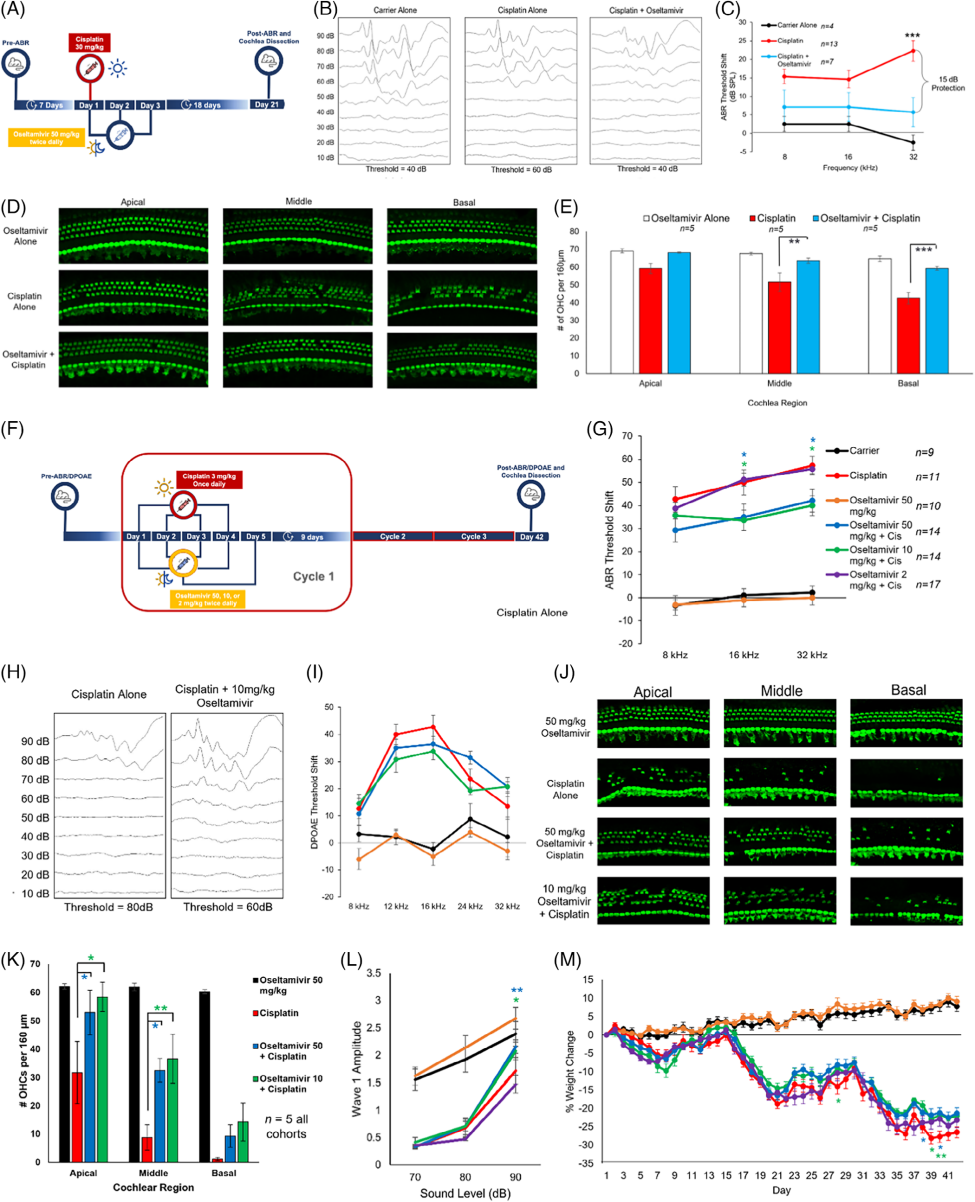
Oseltamivir protects mice from cisplatin‐induced hearing loss and outer hair cell (OHC) loss after a single, high dose of cisplatin, and protects mice from cisplatin‐induced hearing loss and OHC death in a clinically relevant, multicycle cisplatin treatment protocol. (A) Treatment schedule in which mice were treated with a single dose of 30 mg/kg cisplatin and 50 mg/kg oseltamivir twice a day, once in morning and once at night, for 3 days. (B) Representative auditory brainstem response (ABR) traces after the treatment protocol in (A) at the 32 kHz frequency of carrier alone, cisplatin alone and cisplatin + oseltamivir‐treated mice. (C) ABR threshold shifts following treatment protocol in (A). Carrier alone (black), cisplatin alone (red) and oseltamivir + cisplatin (blue). Data shown as means ± SEM, compared to cisplatin alone by two‐way analysis of variance (ANOVA) with Bonferroni post hoc test. ^***^ *p* < .001. (D) Representative whole mount cochlear images stained with myosin VI following treatment protocol in (A). Cochlear whole mount sections from the apical, middle and basal regions were imaged. (E) Quantification of images shown in (D). The number of OHCs per 160 µm was counted per sample in each region. Oseltamivir alone (white), cisplatin alone (red) and oseltamivir + cisplatin (blue). (F) Treatment protocol where mice were treated with 3 mg/kg in the morning for 4 days and then treated with 50, 10 or 2 mg/kg oseltamivir in the morning and night for 5 days. A 9‐day recovery period occurred and then this cycle was repeated two more times for a total of three cycles. (G) ABR threshold shifts following protocol in (F). (H) Representative ABR traces after the treatment protocol in (F) at the 32 kHz frequency of cisplatin alone and cisplatin + 10 mg/kg oseltamivir treated mice. (I) Distortion product otoacoustic emission (DPOAE) threshold shifts following treatment protocol in (F). Carrier (black), oseltamivir alone (orange), cisplatin alone (red), 50 mg/kg oseltamivir + cisplatin (blue), 10 mg/kg oseltamivir + cisplatin (green) and 2 mg/kg oseltamivir + cisplatin (purple). Data shown as means ± SEM, compared to cisplatin alone by two‐way ANOVA with Bonferroni post hoc test. *n* = 9–17. (J) Representative whole mount cochlear images stained with myosin VI following treatment protocol in (F). *n* = 5 (K) Quantification of OHC counts per 160 µm of images shown in (J). Oseltamivir alone (black), cisplatin alone (red), 50 mg/kg oseltamivir + cisplatin (blue) and 10 mg/kg oseltamivir + cisplatin (green). Data shown as means ± SEM, compared to cisplatin alone by one‐way ANOVA with Bonferroni post hoc test. ^*^ *p* < .05, ^**^ *p* < .01. (L) ABR wave 1 amplitude of different treatment groups in (G) at the 16 kHz region. (M) Percent weight loss of all treatment groups following treatment protocol in (F). Mice were weighed everyday throughout the 42‐day treatment protocol. Data shown as means ± SEM, compared to cisplatin alone by one‐way ANOVA with Bonferroni post hoc test. ^*^ *p* < .05, ^**^ *p* < .01. *n* = 9–17 mice.

Oral oseltamivir therapy protects against noise‐induced ABR threshold shifts and cochlear synaptopathy. Mice, females or males, who received 100 mg/kg oseltamivir phosphate 24 h after noise exposure (Figure 3A) exhibited significantly reduced ABR threshold shifts relative to carrier‐treated mice (Figure 3B–D). Mice that received 50 mg/kg oseltamivir had significant protection at 8 kHz (Figure 3E). No ABR protection was observed in the 10 mg/kg oseltamivir treatment group (Figure 3F). No treatment group exhibited protection from DPOAE threshold shifts (Figure 3G–I). Three‐day oseltamivir treatment was sufficient for maximum otoprotective effects when initiated up to 24 h after 100 dB, 2‐h noise exposure, but no protection was achieved with 106 dB, 2‐h noise insult (Figure S1).

**FIGURE 3:**
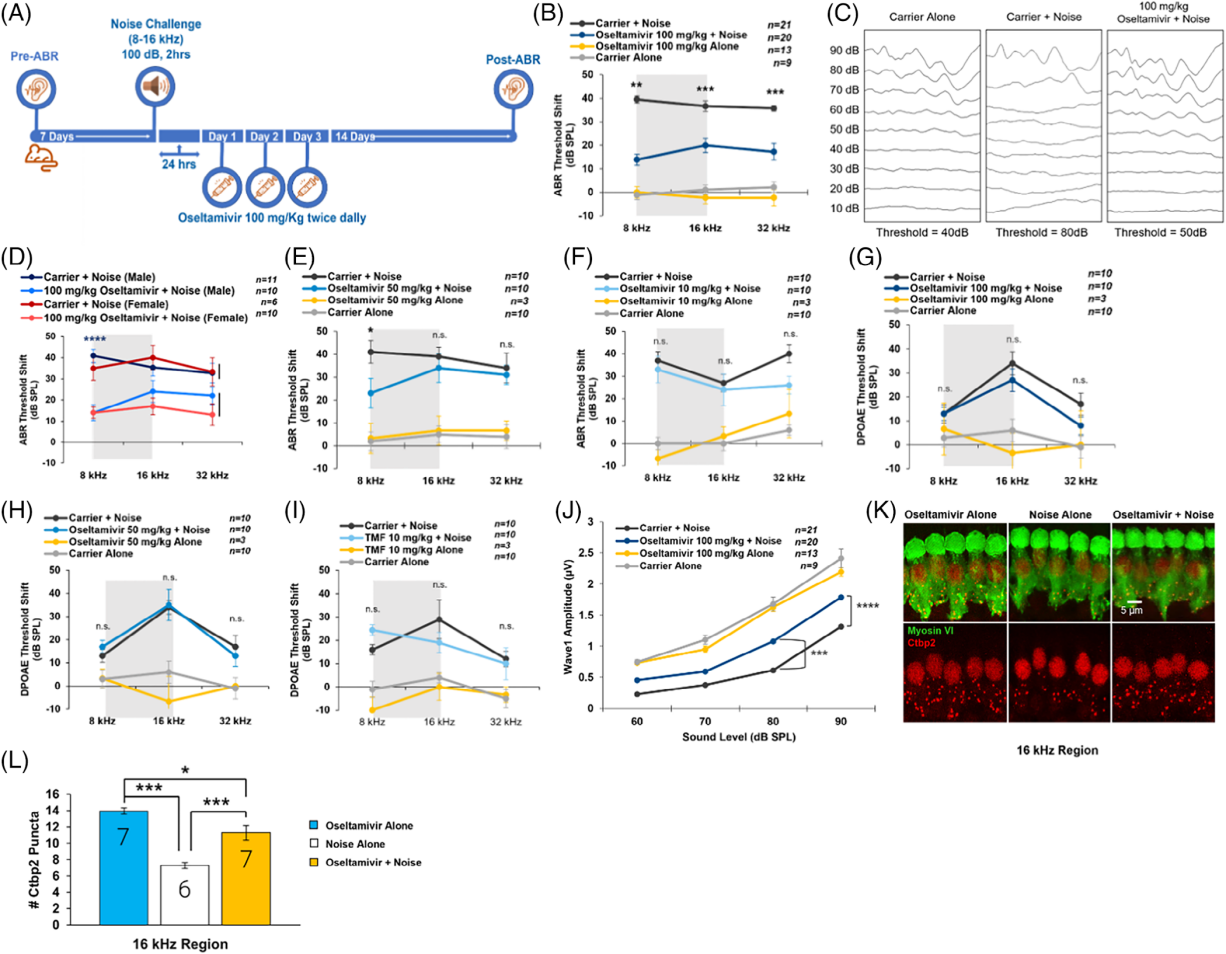
The 100 and 50 mg/kg oral oseltamivir therapy protects against noise‐induced auditory brainstem response (ABR) threshold shift, as well as the morphology and function of the auditory nerve synapse but does not rescue distortion product otoacoustic emission (DPOAE). (A) Schematic of the treatment schedule. (B) The 100 mg/kg oseltamivir treatment significantly protected hearing in noise‐exposed mice with an average reduction of ABR threshold shift of 20−25 dB sound pressure level (SPL) at 8, 16 and 32 kHz. Data shown as means ± SEM compared to carrier + noise by two‐way analysis of variance (ANOVA) with Bonferroni post hoc test. ^**^ *p* < .01, ^***^ *p* < .001. (C) Representative ABR traces after the treatment protocol in (A) at the 16 kHz frequency of carrier alone, carrier + noise alone and noise + oseltamivir‐treated mice. (D) Results in (B) broken down by animal sex. (E) Mice treated with 50 mg/kg oseltamivir exhibited significantly lower ABR threshold shifts at 8 kHz when compared to noise‐exposed mice treated with carrier alone. Data shown as means ± SEM compared to carrier + noise by two‐way ANOVA with Bonferroni post hoc test. ^*^ *p* < .05. (F) No protection was observed with 10 mg/kg oseltamivir. (G–I) DPOAE threshold shifts were also measured for mice treated with 100, 50 and 10 mg/kg, respectively; no significant protection was observed. Data shown as means ± SEM compared to carrier + noise by two‐way ANOVA with Bonferroni post hoc test. (J) ABR wave 1 amplitude for 100 mg/kg oseltamivir versus carrier treated mice, 16 kHz. (K and L) Quantification and imaging of Ctbp2 (auditory synapse) puncta in treated and untreated mice. Data shown as means ± SEM compared to carrier + noise by two‐way ANOVA with Bonferroni post hoc test. ^*^ *p* < .05, ^***^ *p* < .001.

Mice treated with 100 mg/kg oseltamivir portrayed significantly higher average ABR wave 1 amplitude at 90 and 80 dB SPL and higher number of Ctbp2 inner hair cell synaptic puncta relative to carrier‐treated noise exposed mice (Figure 3J–L).

Oseltamivir cisplatin otoprotection is partially mediated through inhibition of pERK protein levels and is associated with reduction in CD45‐positive immune cells in the cochleae of noise‐exposed mice. Given the known binding of oseltamivir to viral neuraminidase, we tested whether the drug would inhibit the activity of mammalian neuraminidases. Cochlear explants were treated with N‐acetyl‐2,3‐dehydro‐2‐deoxyneuraminic acid (DANA), a pan‐selective mammalian neuraminidase inhibitor, or zanamivir, an older antiviral drug that has greater off‐target affinity for mammalian neuraminidases than oseltamivir (Figure 4A–D).^7^ Neither DANA nor zanamivir cotreatments reduced OHCs death relative to cisplatin alone (Figure 4A–D), indicating that oseltamivir's otoprotection is not mediated through neuraminidase inhibition.

**FIGURE 4:**
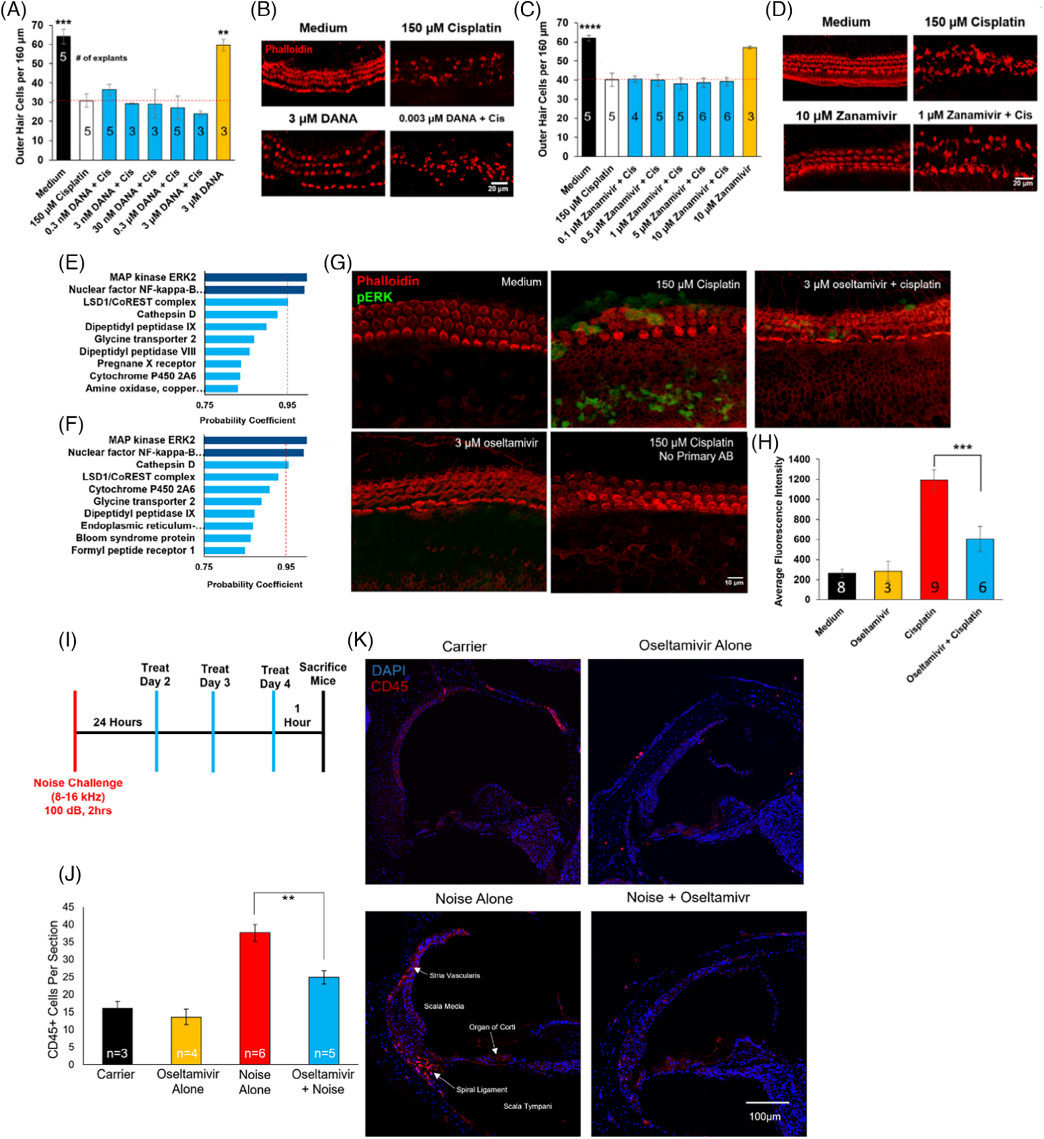
Molecular targets for oseltamivir in otoprotection and anti‐inflammatory role in mice: treatment with other neuraminidase inhibitors fails to mitigate outer hair cell (OHC) loss in cisplatin‐treated murine cochlear explants, indicating oseltamivir's otoprotective qualities are mediated through non‐neuraminidase targets as pERK and inflammation. (A and B) Dose–response relationship between 2,3‐dehydro‐2‐deoxy‐N‐acetylneuraminic acid (DANA) and OHC count in cisplatin‐treated murine cochlear explants; in (B), representative confocal images. DANA is a known inhibitor of mammalian neuraminidases NEU1‐4 (IC50 = 143, 43, 61 and 74 nM, respectively). (C and D) Dose–response relationship between zanamivir and OHCs count in cisplatin‐treated explants; in (D), representative confocal images. Zanamivir is an inhaled influenza neuraminidase inhibitor reported to weakly inhibit mammalian neuraminidases NEU1‐4 (IC50 = 2700, 16.4, 6.8 and 487 µM, respectively). Data shown are means ± SEM, ^**^ *p* < .01, ^***^ *p* < .001 compared to cisplatin alone (white) by one‐way analysis of variance (ANOVA) with Bonferroni post hoc test. (E) Top 10 targets for the pro‐drug, oseltamivir phosphate, predicted by drug target prediction server SuperPred. (F) Top 10 targets predicted by SuperPred for the antiviral active metabolite, oseltamivir carboxylate. (G) Representative confocal images of cochlear explants immunostained with antibody anti‐pERK1/2 (green) and phalloidin (red) following treatment with medium alone, 150 µM cisplatin or 3 µM oseltamivir phosphate + 150 µM cisplatin. (H) Mean corrected total cell fluorescence (CTCF) of pERK1/2 expressing cells in explants following treatment with medium alone, 150 µM cisplatin in medium or 3 µM oseltamivir phosphate in combination with 150 µM cisplatin. Data shown as means ± SEM compared to cisplatin by one‐way ANOVA with Bonferroni post hoc test. ^***^ *p* < .001. (I) Noise exposure and treatment schedule. Mice were exposed to 100 dB noise for 2 h (8–16 kHz) and treated with oseltamivir for 3 days starting 24 h following noise exposure. Cochleae were collected 4 days post‐noise insult. (J) Representative confocal images of cochlear cryosections stained with CD45 (red) and DAPI (blue). (K) Quantification of CD45‐positive cells per cochlear section. Four treatment groups are carrier alone (black), oseltamivir alone (yellow), noise + carrier (red) and oseltamivir + noise (blue). Data shown as means ± SEM, ^**^ *p* < .01 compared to noise alone by one‐way ANOVA with Bonferroni post hoc test. *n* = 3–6 mice.

Next, we submitted the chemical structures of the pro‐drug, oseltamivir phosphate, and its active metabolite, oseltamivir carboxylate, to the drug target prediction server based on binding data, SuperPRED.^8^ ERK2 and NF‐kB p105 were top hits for both compounds at a probability coefficient exceeding .95 (Figure 4E,F). In cochlear mouse explants, mean pERK fluorescence was significantly reduced with cotreatment of 3 µM oseltamivir phosphate compared to cisplatin alone (Figure 4G,H).

To test whether oseltamivir decreases inflammation following noise exposure, mice were exposed to 100 dB SPL noise for 2 h and their cochleae were collected 4 days post‐exposure (Figure 4I). Noise‐exposed mice had a significantly higher average of CD45‐positive cells per cochlear section (37) compared to non‐noise exposed carrier (16) or oseltamivir (14) treated mice. Mice cotreated with 100 mg/kg oseltamivir for 3 days twice daily had a significant reduction in the number of CD45‐positive cells per cochlear section (25) compared to noise alone mice (Figure 4J,K).

Oseltamivir is a widely used antiviral drug with a good safety profile.^9^ Here, we measured significant protection from hearing loss with a dose of 10 mg/kg given twice a day, which is 66% of the mouse equivalent of the standard adult influenza dose.^10^ These results demonstrate promising preclinical data that oseltamivir can be repurposed to protect against cisplatin and noise‐induced hearing loss.

## AUTHOR CONTRIBUTIONS

Tal Teitz, Duane Currier, Jian Zuo and Taosheng Chen designed and performed the cell‐based screens. Matthew A. Ingersoll and Richard D. Lutze performed the in vivo cisplatin experiments. Emma J. Sailor‐Longsworth and Regina G. Kelmann performed the in vivo noise exposure experiments. Matthew A. Ingersoll performed cochlear dissections, outer hair cell and Ctbp2 counts. Emma J. Sailor‐Longsworth, Matthew A. Ingersoll and Kristina Ly performed cochlear explants experiments. Emma J. Sailor‐Longsworth, Regina G. Kelmann, Matthew A. Ingersoll and Richard D. Lutze analysed ABR and DPOAE data. Richard D. Lutze and Regina G. Kelmann performed and imaged the cochlear CD45‐stained cryosections. Richard D. Lutze performed the cisplatin interference testing in tumour cell lines. Emma J. Sailor‐Longsworth, Matthew A. Ingersoll, Richard D. Lutze, Regina G. Kelmann, Kristina Ly and Tal Teitz were involved in the analysis of data and the design of the study. Emma J. Sailor‐Longsworth, Richard D. Lutze, Kristina Ly, Regina G. Kelmann, Matthew A. Ingersoll and Tal Teitz wrote the manuscript with input from all authors.

## CONFLICT OF INTEREST STATEMENT

T.T. and J.Z. are inventors of provisional patent applications filed for the use of oseltamivir in hearing protection #18/129267, #18/106918, and are co‐founders of Ting Therapeutics LLC. All other authors declare that they have no competing interests.

## FUNDING INFORMATION

The research was funded by the following grants: Department of Defense Award #W81XWH‐21‐1‐0696 (grant RH200032), LB 506 Award from Nebraska State, Department of Health and Human Services, Cancer and Smoking Disease Research Program, National Institutes of Health NIDCD (grant 1R01DC018850) and American Hearing Research Foundation 2020 grant to Tal Teitz. This investigation was conducted in facilities constructed with support from Research Facilities Improvement Program (G20 RR024001‐01) from the National Center for Research Resources, NIH. The research was partially conducted at the Auditory and Vestibular Technology Core at Creighton University, Omaha, NE (RRID: SCR_023866). This facility is supported by the Creighton University School of Medicine and grants GM103427 and GM139762 from the National Institute of General Medical Science (NIGMS), a component of the National Institutes of Health (NIH). IBIF was constructed with support from grants from the National Center for Research Resources (RR016469) and the NIGMS (GM103427). This is manuscript #1076 from The Scintillon Research Institute. This investigation is solely the responsibility of the authors and does not necessarily represent the official views of the National Center for Research Resources, NIGMS or NIH.

## Supporting information

Supporting Information

## Data Availability

All data needed to evaluate the conclusions in the paper are present in the paper and/or Supporting Information.
