# Supplementary material for: Oseltamivir (Tamiflu), a commonly prescribed antiviral drug, mitigates hearing loss in mice
Source: Clin Transl Med. 2024 Aug 12;14(8):e1803. doi: 10.1002/ctm2.1803 (PMC11318337; doi:10.1002/ctm2.1803)
Supplement: Supplementary file 1 — Supporting Information [file CTM2-14-e1803-s001.docx]

**Supplemental Information**


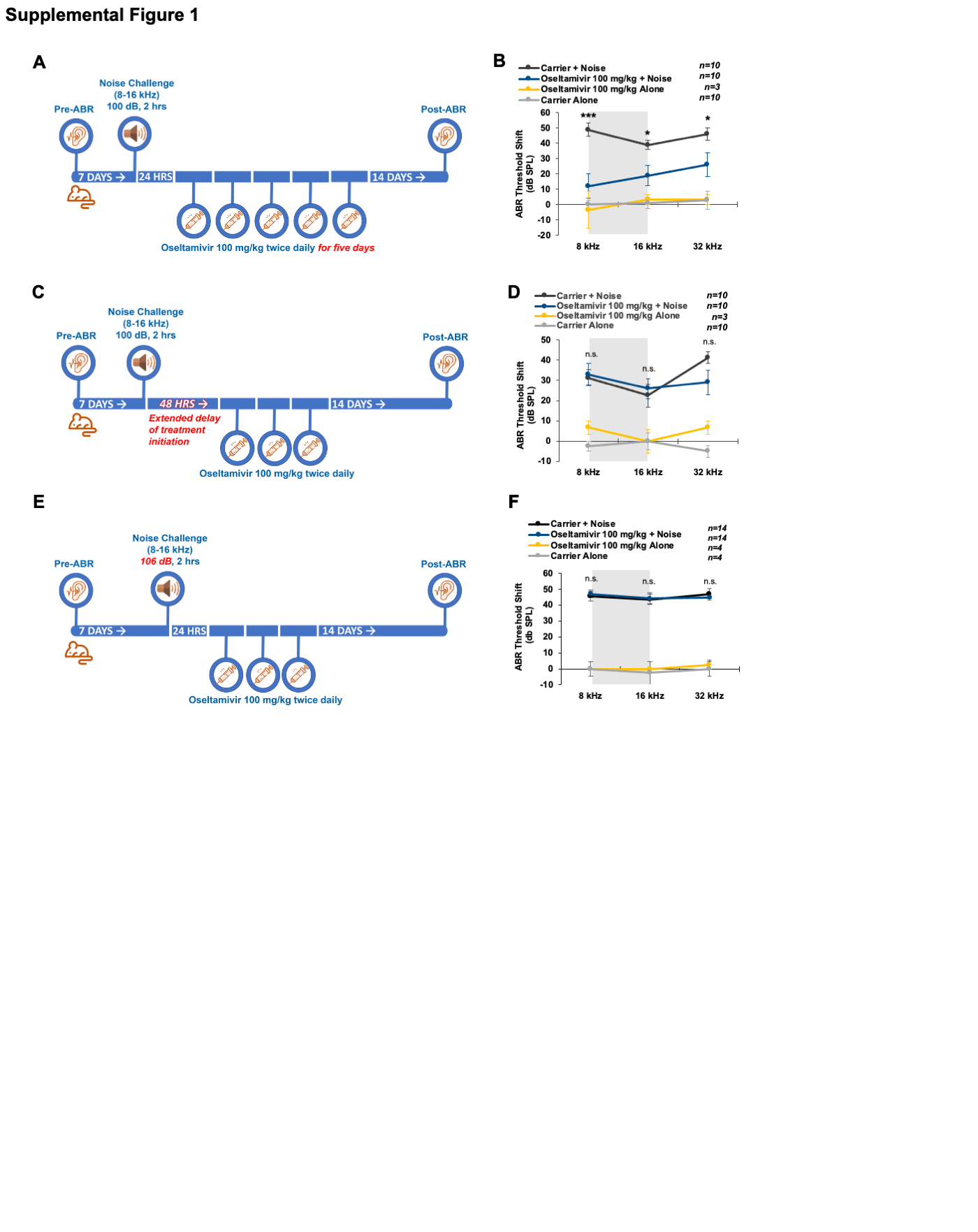


**Supplemental Figure 1: 100 mg/kg oral oseltamivir treatment twice daily is protective against ABR threshold shifts when started 24 hours after noise and continued at least for three days, but not when started 48 hours post-noise challenge or following 106 dB noise exposure. (A)** Schematic diagram of extended treatment schedule. Oseltamivir was initially tested at 100 mg/kg twice daily, beginning 24 hours post noise challenge and continuing for three days. The first alternative treatment schedule involved extending treatment to 5 days**. (B)**  ABR threshold shifts for mice treated with oseltamivir for an extended duration of five days. **(C)** Schematic diagram of delayed treatment schedule (48 hrs post-noise exposure). **(D)** ABR threshold shifts for mice treatment with oseltamivir (100 mg/kg) beginning 48 hours post-noise exposure. **(E)** Schematic diagram illustrating treatment schedule following a noise exposure of increased volume (from 100 to 106 dB). Mice were treated as in Figure 5A with no adjustment made in treatment timing or duration. **(F)** ABR threshold shifts for mice treated with 100 mg/kg oseltamivir phosphate following 106 dB noise challenge. Data in (B),(D) and (F) shown as means ± SEM compared to carrier + noise by two-way ANOVA with Bonferroni post-hoc test. *p<0.05, ***p<0.001.

**Materials and Methods**

**Ethics statement**

All animal procedures were approved by the Institutional Animal Care and Use Committee of Creighton University (IACUC) in accordance with policies established by the Animal Welfare Act (AWA) and Public Health Service (PHS).

**Mouse models**

All noise exposure experiments, and the single dose cisplatin protocol were conducted using FVB/NJ mice acquired from Jackson Laboratory (Bar Harbor, Maine, USA) and descendants bred in the Creighton University Animal Resource Facility (ARF). For the multicycle cisplatin mouse model, 8-week-old CBA/CaJ mice were purchased from Jackson Laboratory and were given one week to acclimate before any experiments began. For all mouse models, anesthesia was performed using 500 mg/kg Avertin (2,2,2-tribromoethanol) delivered via intraperitoneal injection. Loss of righting reflex and pedal reflex (as assessed via toe pinch) were used as benchmarks for determining whether adequate levels of anesthesia had been achieved prior to measurement of ABR and DPOAE. Mice were randomly assigned to all treatment groups with equal numbers of female and male mice included per group.

**High-throughput screen for identifying candidate drugs protective from cisplatin-induced cell death in murine inner ear cell line**

Oseltamivir phosphate was identified as potentially otoprotective via the cell-based high-throughput screen described^1-3^. Briefly, cells of the murine inner ear cell HEI-OC1 were plated at a previously optimized concentration of 1600 cells per well and tested against 50 μM cisplatin for apoptotic activity via the Caspase 3/7-Glo assay. Approximately 1,300 FDA approved small molecule compounds were tested in the screen where oseltamivir phosphate was identified as a top hit. The compounds were dissolved in DMSO prior to addition to medium with final DMSO concentration in medium kept below 0.5%. Pifithrin-α was employed as a positive reference compound with an IC_50_ of 7.7 μM for inhibition of cisplatin-induced caspase-3 cleavage. Hits were defined as compounds that reduced caspase-3/7 activity by 50% or more when co-administered with 50 μM cisplatin.

**Cochlear explants**

Cochleae were collected from P3 FVB/NJ mice^1,3^. Immediately following collection, cochleae were plated in six-well plates (2-3 explants/well) containing 1 ml media (growth medium DMEM (12430-054,Gibco Life Technologies) combined with 1% FBS (16000-044, Gibco Life Technologies), B-27 supplement (200 μl/500 ml; 17504-44, Gibco Life Technologies), N-2 supplement (100 μl/500 ml; 17502-048, Gibco Life Technologies), and ampicillin (50 μg/ml; A5354-10ML, Sigma-Aldrich). Cochlear explants were then incubated for 24 hours at 37°C and 5% CO_2_. Following 24 hours, the explants were pretreated with fresh media with or without the compounds of interest (oseltamivir phosphate, oseltamivir carboxylate, zanamivir, or DANA) at various concentrations and then incubated at 37°C and 5% CO_2_ for an additional hour. Media was then replaced with fresh growth media with or without the compounds of interest in addition to 150 μM cisplatin (479306, Sigma-Aldrich), after which the explants were again incubated for a final 24 hours at 37°C and 5% CO_2_. Cochlear explants were then fixed in 4% paraformaldehyde in 1x PBS for a minimum of one hour. The explants were stained for F-actin with Alexa Fluor 568 Phalloidin (1:400 dilution, A12380, Thermo Fisher Scientific) or antibody anti-pERK1/2 (Thr^202^/Tyr^204^, 1:400 dilution, 9101, Cell Signaling Technology) and imaged with a confocal microscope (LSM 700, Zeiss). Outer hair cell number were counted per 160 μm in the cochlear middle turn region.

**Drug preparation and administration**

Oseltamivir phosphate powder was acquired from MedChemExpress (HY-17016) and dissolved in a carrier solution of 10% DMSO, 40% PEG 300, 5% Tween-80 and 45% saline for administration by oral gavage. Doses of 100, 50, 10, or 2 mg/kg were administered to the mice at the respective times for each individual experiment. Oseltamivir carboxylate, DANA and zanamivir powder was purchased from MedChemExpress (HY-13318, HY-125798, HY-13210) and dissolved in DSMO prior to serial dilution in media for the cochlear explant experiments.

**Auditory brainstem response**

ABR waveforms recordings were collected in mice anesthetized as described previously^1,3-6^. Recordings occurred in a sound booth (Industrial Acoustic Company) via three subdermal needle electrodes placed medially down the skull, below the pinna of the left ear, and at the base of the tail. Responses were fed into a low-impedance Medusa digital biological amplifier system (RA4L; TDT; 20-dB gain). At 8, 16, and 32 kHz, mice were exposed to auditory stimuli starting at 90 dB; to determine the minimum threshold dB-SPL for each frequency tested, the stimulus intensity was then reduced in 10 dB intervals until a minimum intensity of 10 dB was reached. ABR waveforms were averaged in response to 500 tone bursts and recorded signals were filtered by a band-pass filter (300 Hz to 3 kHz). ABR threshold was defined as the lowest dB tested where at least 3 of the 5 characteristic waveform peaks were still present. For all experiments, mice with baseline ABR thresholds equaling or exceeding 50 dB at any frequency were considered to have pre-existing hearing loss and thus were excluded from further experimental use. Furthermore, all ABR threshold readings were independently validated by 2 to 3 additional readers blinded to the treatment group assignments of the mice. Threshold shifts were calculated by subtracting pre-exposure thresholds from post-exposure thresholds for each frequency. ABR wave 1 amplitude were measured at 16 kHz in post-exposure recordings and were defined as the difference between the wave 1 peak and the noise floor for a given ABR trace.

**Distortion product otoacoustic emission**

DPOAE recordings were collected from mice anesthetized as previously described^5,6^. DPOAE recordings were collected in a sound booth (Industrial Acoustic Company) using the ER10B+ microphone system, with ear tip and speaker tubes placed in the left ear canal such that the tympanic membrane remained unobstructed. Data collection and analysis was performed on the TDT RZ6 workstation running BioSigTZ software (Tucker-Davis Technologies). DPOAE was measured at 8, 16, and 32 kHz with an f2/f1 ratio of 1.2. Tone 1 was *.909 of the center frequency and tone 2 was *1.09 of the center frequency. DPOAE data was recorded every 20.97 milliseconds and averaged 512 times at each intensity level and frequency tested. For each frequency tested, the initial stimulus intensity was 90 dB and was then decreased in increments of 10 dB until a minimum intensity of 10 dB was reached. The DPOAE threshold was defined as the lowest dB tested at a given frequency to exhibit an emission above the noise floor. DPOAE threshold shift was defined as equaling the difference of the baseline DPOAE threshold and the post-experimental DPOAE threshold for each frequency tested.

**Traumatic noise exposure model and treatment**

Baseline hearing tests (ABR and DPOAE) were performed as previously described on 6–8-week-old FVB/NJ mice one week in advance of noise exposure^1,3-6^. To model noise-induced hearing loss and acoustic trauma, the mice were exposed to 100 dB SPL noise over a 8-16 octave band for two hours. The noise challenge took place within a sound-tight acrylic chamber (custom-built; Creighton University physics machining shop) which housed a top-mounted JBL speaker positioned above and facing down towards the mice.  To ensure all mice received equivalent noise coverage from the speaker, the sound chamber was equipped with a metal wire enclosure consisting of ten individual compartments; these compartments were distributed in a circle, with each compartment equidistant from the other as well as from the top-mounted speaker. The sound stimulus was generated using a System RZ6 (Tucker-Davis Technologies) workstation and amplified through a 75-A power amplifier (Crown). Prior to each noise exposure challenge, sound pressure level was calibrated using an NSRT-mk3 microphone (convergence instruments) to confirm each compartment within the enclosure was within 0.5 dB of 100 dB and ensure equal noise exposure for all mice. Treatment with oseltamivir phosphate (10, 50, or 100 mg/kg) or carrier began 24 hours after the cessation of noise exposure, and was continued twice daily for 3-5 days including day of first treatment; the efficacy of treatment with 100 mg/kg oseltamivir phosphate when initiated 48 hours was also studied. Post-exposure ABR and DPOAE measurements were then collected 14 days after noise exposure, when mice were 8-10 weeks of age.

**Single high dose cisplatin treatment model**

Pre-experimental ABR were performed on 6–8-week-old FVB mice. One week after ABR testing, mice were treated with 50 mg/kg oseltamivir phosphate 45 minutes before a 30 mg/kg cisplatin intraperitoneal injection in the morning. Mice were then treated with oseltamivir phosphate again in the evening. Oseltamivir phosphate was administered twice a day for three total days. 21 days after the single cisplatin injection, post-experimental ABR were performed, and cochleae were harvested and put in 4% PFA solution. One day before cisplatin injection, mice received 1 mL of saline by subcutaneous injection and were given 1 mL of saline twice a day throughout the protocol until body weight started to recover. The cages of cisplatin treated mice were placed on heating pads until body weights began to recover. Food pellets dipped in DietGel Boost® were placed on the cage floor of cisplatin-treated mice. DietGel Boost® (72-04-5022 Clear H2O) is a high calorie dietary supplement that provides extra calorie support for mice. The investigators and veterinary staff carefully monitored for changes in overall health and activity that may have resulted from cisplatin treatment.

**Multi-cycle cisplatin treatment model**

Pre-experimental ABR were performed on 9-week-old CBA/CaJ mice with DPAOE performed when mice were 10 weeks old. Once mice were 12 weeks old, the 6-week cisplatin and oseltamivir phosphate treatment regimen began. Oseltamivir phosphate (50, 10, or 2 mg/kg/bw) was administered via oral gavage 1 hour before 3 mg/kg cisplatin was administered to mice via intraperitoneal injection in the morning. Mice were then treated with oseltamivir phosphate or carrier again in the evening. Mice were treated with cisplatin once a day for 4 days and oseltamivir phosphate twice a day for 5 days with a 9-day recovery period in which no drugs were administered to the mice. This cycle was repeated two more times for a total of 3 cycles. Mice were treated with 3 mg/kg cisplatin for a total of 12 days (4 days per cycle with 3 cycles) and oseltamivir phosphate for a total of 15 days (5 days per cycle with 3 cycles). Immediately after the completion of cycle 3 (42 days after the first cisplatin injection), post-experimental ABR were performed with DPOAE performed one week after ABR. Cochleae were when harvested and put in 4% PFA. Cisplatin treated mice were injected by subcutaneous injection twice a day with 1 mL of warm saline to ameliorate dehydration. This continued until body weight started to recover. The cages of cisplatin-treated mice were placed on heating pads throughout the duration of the experiment until mice began to recover after the 3rd treatment cycle of the protocol. Food pellets dipped in DietGel Boost® were placed on the cage floor of cisplatin-treated mice. The investigators and veterinary staff carefully monitored for changes in overall health and activity that may have resulted from cisplatin treatment.

**Outer hair cell counts**

Cochleae from adult mice were prepared and examined as described previously ^1, 3-7^. Cochleae samples were immunostained with anti-myosin VI (1:400; 25-6791, Proteus Bioscience) with secondary antibodies purchased from Invitrogen coupled to anti-rabbit Alexa Fluor 488 (1:400; A11034). All images were acquired with a confocal microscope (LSM 700 or 710, Zeiss). Outer hair cell counts were determined by the total amount of OHCs in a 160 µm region. Counts were determined for the 8, 16, and 32 kHz regions. Cochleae from each experimental group were randomly selected to be imaged for outer hair cell counts.

**Ctbp2 staining for quantification of auditory nerve puncta**

Cochlear dissections were performed at the conclusion of noise exposure studies. After fixation in 4% PFA, otic capsules underwent brief decalcification for 30 minutes in a 120 mM EDTA solution. The organs of corti were then dissected from the otic capsule and co-stained with anti-Ctbp2 antibodies (1:800; 612044, BD Transduction) and anti-myosin VI antibodies (1:400; 25-6791, Proteus Biosciences) overnight at 4ºC. Goat anti-rabbit Alexa Fluor 488 (1:400; A11034) and goat anti-mouse Alexa Fluor 647 (1:800; A32728) were purchased from Invitrogen as secondary antibodies. Images of the 16 kHz regions of each sample were collected upon the LSM 700 confocal microscope (Zeiss) at a 63x objective. Maximum intensity projections were obtained using ZEN BLACK software (Zeiss) with Ctbp2 puncta counted in ImageJ. 12-18 inner hair cells were visible per image and the average number of Ctbp2 puncta per sample was calculated as equaling the total number of puncta divided by the total number of inner hair cells visible. Cochleae used for Ctbp2 puncta quantification were obtained from 6-7 randomly selected mice per treatment group.

**Cochlear cryosectioning and CD45 staining**

FVB mice aged 6-8 weeks old were exposed to 100 dB SPL noise (8-16 kHz octave band) for 2 hours. Mice were either treated with carrier alone or 100 mg/kg oseltamivir twice a day for 3 days beginning 24 hours after noise exposure. Mice were then sacrificed 1 hour after the last drug treatment which was approximately 4 days following noise exposure. Cochleae were extracted from mice and placed in 4% PFA for 2-3 days. Cochleae were then decalcified in 120 nM EDTA for 2-3 days. Following decalcification, cochleae were transferred to a 30% sucrose solution and kept at 4°C overnight. The next day, samples were put into a solution of 30% sucrose and OCT compound (4583; Sakura) for 4 hours at 4°C. Samples were then placed in OCT compound overnight at 4°C. The next day, cochlear tissues were oriented in within cryomolds containing OCT compound and frozen on dry ice. Frozen tissues were cut with 10µm thickness. captured on glass slides and allowed to dry for several hours.

Cochlear cryosections were then blocked and permeabilized in a solution of 5% FBS and 0.2% triton X-100 in PBS. Tissues were stained overnight at 4°C with mouse CD45 antibody (1:50; Af114, R&D Systems). The next day, tissues were stained for one and a half hours with Alexa Fluor 568 donkey anti-goat (1:400; A11057, Invitrogen) and DAPI (1:1000; D1306, Invitrogen) to counterstain nuclei. Tissues were mounted in Fluoromount-G (00-4958-02, Invitrogen) and imaged using a Zeiss 700 upright confocal microscope. Post-acquisition images were analyzed using the IMARIS imaging software and automatically quantified following intensity thresholding. CD45 positive cells were then cross-checked manually to ensure positive CD45 cells were co-stained with DAPI.

**Statistical analysis**

Statistical analysis was performed in Prism (GraphPad Software). Two-way analysis of variance (ANOVA) or one-way ANOVA with Bonferroni post hoc test was used to determine mean difference and statistical significance.

**Supplemental References**

1. Ingersoll MA, Malloy EA, Caster LE, Holland EM, Xu Z, Zallocchi M, Currier D, Liu H, He DZZ, Min J, Chen T, Zuo J, Teitz T. BRAF inhibition protects against hearing loss in mice. Sci Adv. 2020 Dec 2;6(49):eabd0561. doi: 10.1126/sciadv.abd0561. PMID: 33268358; PMCID: PMC7821884.
2. Teitz T, Goktug AN, Chen T, Zuo J. Development of Cell-Based High-Throughput Chemical Screens for Protection Against Cisplatin-Induced Ototoxicity. Methods Mol Biol. 2016;1427:419-30. doi: 10.1007/978-1-4939-3615-1_22. PMID: 27259939.
3. Teitz T, Fang J, Goktug AN, Bonga JD, Diao S, Hazlitt RA, Iconaru L, Morfouace M, Currier D, Zhou Y, Umans RA, Taylor MR, Cheng C, Min J, Freeman B, Peng J, Roussel MF, Kriwacki R, Guy RK, Chen T, Zuo J. CDK2 inhibitors as candidate therapeutics for cisplatin- and noise-induced hearing loss. J Exp Med. 2018 Apr 2;215(4):1187-1203. doi: 10.1084/jem.20172246. Epub 2018 Mar 7. PMID: 29514916; PMCID: PMC5881471.
4. Ingersoll MA, Lutze RD, Kelmann RG, Kresock DF, Marsh JD, Quevedo RV, Zuo J, Teitz T. KSR1 Knockout Mouse Model Demonstrates MAPK Pathway's Key Role in Cisplatin- and Noise-induced Hearing Loss. J Neurosci. 2024 May 1;44(18):e2174232024. doi: 10.1523/JNEUROSCI.2174-23.2024. PMID: 38548338; PMCID: PMC11063821.
5. Ingersoll MA, Lutze RD, Pushpan CK, Kelmann RG, Liu H, May MT, Hunter WJ, He DZ, Teitz T. Dabrafenib protects from cisplatin-induced hearing . loss in a clinically relevant mouse model. JCI Insight. 2023 Dec 22;8(24):e171140. doi: 10.1172/jci.insight.171140. PMID: 37934596; PMCID: PMC10807719.
6. Lutze RD, Ingersoll MA, Thotam A, Joseph A, Fernandes J, Teitz T. ERK1/2 Inhibition via the Oral Administration of Tizaterkib Alleviates Noise-Induced Hearing Loss While Tempering down the Immune Response. International Journal of Molecular Sciences. 2024; 25(12):6305. <https://doi.org/10.3390/ijms25126305>
7. Yamashita T, Fang J, Gao J, Yu Y, Lagarde MM, Zuo J. Normal hearing sensitivity at low-to-middle frequencies with 34% prestin-charge density. PLoS One. 2012;7(9):e45453. doi: 10.1371/journal.pone.0045453. Epub 2012 Sep 21. PMID: 23029017; PMCID: PMC3448665.
